# Effects of affectively-loaded childhood-related photos from the IAPS on the induction of involuntary autobiographical memories in young and older adults

**DOI:** 10.3389/fpsyg.2023.1266758

**Published:** 2024-01-11

**Authors:** Beatriz Navarro, María Verónica Jimeno, Luz Fernández-Aguilar, Marta Nieto, Abel Toledano-González, María José Cantero, Laura Ros, José Miguel Latorre

**Affiliations:** ^1^Unidad de Psicología Cognitiva Aplicada, Instituto de Discapacidades Neurológicas, Universidad de Castilla-La Mancha, Albacete, Spain; ^2^Departamento de Psicología, Facultad de Medicina de Albacete, Universidad de Castilla-La Mancha, Albacete, Spain; ^3^Departamento de Psicología, Facultad de Derecho, Universidad de Castilla-La Mancha, Albacete, Spain; ^4^Departamento de Psicología, Facultad de Ciencias de la Salud, Talavera de la Reina, Toledo, Spain; ^5^Departamento de Psicología Evolutiva y de la Educación, Universitat de València, Valencia, Spain

**Keywords:** aging, autobiographical memory, emotions, photos, IAPS, emotion induction

## Abstract

**Objective:**

Aging produces changes in emotional reactivity and the retrieval of autobiographical memories. The main aim of this study was to assess age-related differences, comparing emotion induction and autobiographical memory recall using photos from the International Affective Picture System (IAPS) that are thematically related to childhood.

**Method:**

A cross-sectional observational study was conducted, with the participation of 327 individuals (168 young adults and 159 older adults) with no cognitive impairment and aged between 18 and 88 years. We showed the participants a set of five pictures from the IAPS, the affective content of which was related to childhood. Two of these were considered to be positive images, two negative and one neutral, according to the valence of these pictures in the literature. The main study variables were the reactions associated with emotional valence or pleasure, arousal and dominance, after viewing the photos, and the autobiographical memories retrieved by the participants.

**Results:**

The younger adults retrieved a larger number of memories than their older counterparts. As regards the responses to the five affective pictures (IAPS) on valence, arousal and dominance (IAPS), statistically significant differences were only found for pictures 2,345 (BlackEye), with a more positive valence in the group of older adults and higher arousal in the young ones, and 2,312 (Mother), with a more positive valence in the group of older persons. A greater number of memories were retrieved for the photos that generated higher levels of pleasure, greater relaxation and greater emotional control.

**Conclusion:**

Of the variables that may be associated with the elicitation of involuntary autobiographical memories, the most significant are age and a positive stimulus.

## 1 Introduction

Aging produces changes in emotional reactivity. Several theories have been proposed to explain these changes. One of these is the socioemotional selectivity theory (SST), which holds that, as people age, their socioemotional goals change and are focused more on intimate, affective relationships, such as those with family and close friends (Carstensen, 2006). The use of affectively-loaded photos is one of the most commonly used techniques in research on emotional reactivity. By comparing young and older adults, the present work seeks to examine how using childhood-related photos from the International Affective Picture System (IAPS) (Lang et al., 2008) impacts emotional response (specifically, reactivity) and autobiographical memory induction. Reactivity, or emotional reaction, refers to the change in response from a non-emotional state (baseline) to the magnitude of response elicited by a potentially emotional stimulus. This response occurs immediately upon presentation of the stimulus and must be differentiated from other components involved in emotional states, such as emotion regulation (Gruber et al., 2011).

Autobiographical Memory (AM), unlike other memories, refers to a knowledge base of personal information that includes specific episodic memories of past events and more conceptual, self-related information. Something that differentiates autobiographical memories from other types of memories is that the former have been experienced in the first person. They are not stories that we have been told or that we have extracted from a particular source, such as literature. Autobiographical knowledge is hierarchically organized across different levels of specificity (Conway and Pleydell-Pearce, 2000). At the general level, we can find extended memories, which are general memories associated with events that last more than a day (e.g., “the time I spent at university”), and categorical memories, which are general, repeated events grouped together in a category (e.g., “summer holidays with my family at the beach”). At the lowest level of the hierarchy are specific AMs, which are personally significant memories associated with a time and place that lasted a day or less than a day (e.g., “the day I passed my driving test”). Difficulty in retrieving specific autobiographical memories is known as overgeneral autobiographical memory (Williams, 2006). The tendency to overgeneralize is more common in older persons, who recall fewer specific events than young adults (Ricarte et al., 2016; Ros et al., 2017).

To understand why it is important to investigate the effect of child-related photos from the IAPS impacts emotional reactivity and autobiographical memory induction, we present a review of research studies where older adults’ perception and retrieval of memories are found to contain more positive than negative information compared to young adults.

As people grow older, they typically remember and attend more to positive information than negative information, while also being more inclined toward emotionally gratifying memory, compared to young adults. Additionally, when shown stimuli of differing affective valences, positive stimuli generate more memories in older adults than in young persons. This phenomenon is known as the positivity effect (Mather and Carstensen, 2005). As an example, in two studies where authors examined age differences in recall and recognition memory for positive, negative and neutral stimuli, using images on a computer screen and a distraction task before the memory task, the number of negative images recalled, compared with positive and neutral ones, decreased with age (Charles et al., 2003). A systematic meta-analysis (*N* = 7,129) examined this preference for positive over negative information between young and older adults. In this meta-analysis, the authors included studies with different stimuli, such as autobiographical memories, advertisements, faces, images or videos, among others. The results indicated that, compared to young adults, older adults attend to, and remember, positive information better than negative information (Reed et al., 2014).

The socioemotional selectivity theory is one of the theories most commonly called upon to explain this effect. It holds that our time horizon affects our goals, especially those related to knowledge acquisition and satisfying emotional needs. When we perceive time as open-ended, our goals are more likely to be preparatory, for example, collecting information, living novel experiences or increasing the depth of our knowledge by, among other means, choosing social relationships that facilitate such goals. When time is perceived as constrained, goals emphasize emotional states, particularly those that trigger well-being (Mather and Carstensen, 2005), and we choose social relationships with close friends or family rather than with people that may provide us with new knowledge. Young people’s goals tend to be more future-oriented, while older adults prioritize present-oriented goals that maximize emotional well-being (Löckenhoff and Carstensen, 2004). Regarding cognitive processing, the SST suggests that, with age, people place greater emphasis on emotionally relevant information and reallocate their processing resources toward the positive aspects of a situation more than the negative ones. The same occurs with memory, such that when emotional goals are prioritized, emotionally relevant information is likely to be remembered more easily (Löckenhoff and Carstensen, 2004). Focusing on autobiographical memory, in a study that asked young and old dyads of different ages to recall a vacation from the past, the young couples’ memories were found to contain more objective information, while their older counterparts retrieved more thoughts and feelings (Gould and Dixon, 1993).

Research on autobiographical memory and aging has typically focused on voluntary recall of memories, although, in everyday life, such memories tend to surface involuntarily or unintentionally (Schlagman et al., 2009). Studies on involuntary autobiographical memory also underline age-related differences, with older people having greater difficulty in retrieving memories (Berntsen and Rubin, 2002; Schlagman et al., 2009), although a large body of literature also reports no age effect (Rubin and Berntsen, 2009).

There are two key issues related to the eliciting of autobiographical memories using pictures, namely, the effect of the reminiscence bump (RB) and the differences in the content of memories depending on the stage of psychosocial development. The RB refers to how, as people age, they generate more memories from the time between the ages of approximately 10 and 30 years. When an individual is asked to freely recall personally significant memories, they tend to date from this period (Munawar et al., 2018). Additionally, memories of childhood and early adolescence are typically associated with family relationships and early affective attachments, such as memories with parents, siblings or family in general, since the memories are frequently generated and maintained in family conversations and relationships (Conway and Pleydell-Pearce, 2000; Conway, 2005; Fivush, 2011; Kim et al., 2020).

Autobiographical memory has frequently been assessed using stimuli in the form of cue words (Ros et al., 2018; Barry et al., 2021), although other methods used to elicit the retrieval of autobiographical memories include stimuli such as images, odors, textures, personal items and sounds (Williams et al., 1999; Hackländer et al., 2019; Van den Hoven et al., 2021; Jakubowski et al., 2023a,b). A study by St-Laurent et al. presented a series of photos to two groups of participants (young adults and older adults), who were asked to retrieve an autobiographical memory thematically related to the image and to rate its vividness. The authors found a differences in the neural correlates of the memory task and a loss of autobiographical memory specificity in the older group (St-Laurent et al., 2011). A more recent study examined the effect of pictorial cues on autobiographical memory in healthy individuals and patients with Alzheimer’s disease, showing higher memory after visual cuing compared to no cuing, in both healthy persons and patients with Alzheimer’s disease. The results led the authors to conclude that pictorial clues may be a powerful tool for eliciting autobiographical memories in both research and clinical practice (El Haj et al., 2020).

Controlled mood induction is necessary to enhance our knowledge of emotions. On this basis, numerous Mood Induction Procedures (MIPs) have been designed for the purpose of inducing positive, negative and neutral mood states in laboratory settings (Fernández-Aguilar et al., 2019). Exposure to pictures with affective content is one of the prominent methods used in MIPs. Specifically, the IAPS is the pictorial database most widely used in the scientific literature (Lang et al., 1998, 2008), with both healthy participants (Britton et al., 2006; Laureanti et al., 2020) and those with mental disorders (Jayaro et al., 2008). In addition, the IAPS has been extensively used in research on emotions in aging, with age differences reported for both positive and negative stimuli (Grühn and Scheibe, 2008). Furthermore, recent studies that have used film clips to induce different emotions have also shown these age-related differences when assessing the response using both self-report measures and physiological emotional reports (Fernández-Aguilar et al., 2020).

### 1.1 Study proposal

Research on autobiographical memory retrieval based on exposure to photos is closely linked to the implementation of reminiscence techniques, using participants’ own photos, generally from childhood or adolescence (Carretero et al., 2020; Fernández-Pérez et al., 2023; Toledano-González et al., 2023). Yet, what happens when older adults and young adults are presented non-personal photos that are thematically associated with childhood and family relationships? Does the presence or absence of childhood family relationships in an image generate greater reactivity in older adults compared to young persons? And what happens with the autobiographical memories associated with the images?

Comparing young and older adults, the present study examines the differences in emotional reactivity and autobiographical memories using a set of photos with affective content related to childhood. Using photos taken from the IAPS rather than personal photographs allows us to better verify the aims of the study since, to compare young and old participants, we presented exactly the same image as a stimulus. It has also been documented in the literature that, when pictures from personal life are unavailable, the selection of images capable of generating positive autobiographical memories is a feasible tool for older adults’ emotion regulation (Carretero et al., 2020). In contrast to previous studies, in the present work, participants were not given instructions to retrieve a memory when they saw the photos, but were asked, after each image, whether viewing the photos had brought to mind any autobiographical memories. Hence, we are dealing with spontaneous or involuntary autobiographical memories.

## 2 Method

### 2.1 Design

The study is observational, analytical and cross-sectional.

### 2.2 Sample

A total of 327 individuals voluntarily participated in the study. We formed two age groups; one comprised 168 young adults (51.38%) aged between 18 and 27, while the other was made up of 159 older persons aged between 65 and 88. The mean age of the young participants was 19.48 (SD = 1.91) and the mean age of their older counterparts was 72.23 (SD = 6.34). Of these, 63.50% of the sample were women and 36.40% were men. In the young adult group, 27.4% of the participants were men, while they accounted for 46.2% of the older adult group, (*p* < 0.001).

The mean length of time in education in the young group was 12.61 years (SD = 0.49) and 9.04 (SD = 5.89) in the older group, *t*(154) = 7.46, *p* < 0.001. Absence of cognitive impairment was tested using Test your Memory (TYM) (Ferrero-Arias and Turrión-Rojo, 2016), yielding a mean of 45.65 (SD = 2.28) and a range of 41 to 49. According to the scale, scores of 40 or below indicate the presence of mild cognitive decline.

Sample power was calculated using the sensitivity test with the G*Power program. For the t-test for independent group comparison, α err prob. = 0.05, power (1-β err prob) = 0.95, sample size group young = 165, sample size group older = 165, non-centrality parameter δ = 3.29, 330 df, critical value of t = 1.64 and minimal effect side d = 0.36. For logistic regression, effect direction = p2 < = p1, α err prob. = 0.05, Pr(Y = 1|X = 1) H0 = 0.2, power (1-β err prob) = 0.95, total sample size = 327, R^2^ other X = 0, normal distribution X, X parm μ = 0, X parm σ = 1, critical value of z = −1,6,448,536, Odds ratio = 0,6,320,602 and actual power = 0.95.

### 2.3 Instruments

The IAPS (Lang et al., 1998) is a battery of 1,000 photos of people, animals objects and landscapes. The images are classified into the categories of affective valence or pleasure, arousal and dominance. The photos are able to elicit both positive (e.g., a group of children playing) and negative emotions (e.g., a murder scene). The battery has been validated in Spanish, with the authors reporting it to be an excellent technical tool for scientific research on emotions in Spanish population (Moltó et al., 1999, 2013). We chose a collection of five pictures representing childhood for use in our study. The slides are tagged with standardized valence values, so that experimental stimuli were classified as negative, neutral or positive in emotional content (Wei et al., 2020). Drawing on the standardized ratings provided with the tool, we used the following pictures: 2299 and 5,831 (positive stimuli); 2,345 and 9,041 (negative stimuli); and 2,312 (neutral stimulus) (Lang et al., 2008).

The Self-Assessment Manikin (SAM) (Bradley and Lang, 1994) is a non-verbal pictorial assessment technique that assesses three aspects of a person’s emotional experience following exposure to a stimulus (in our case, the pictures from the IAPS): emotional valence or pleasure, arousal and dominance. It is a self-report questionnaire, in which, for the dimension of pleasure, the scale ranges from a smiling, happy figure to frowning, unhappy figure. For arousal, the scale ranges between an excited, wide-eyed figure to a relaxed, sleepy figure. Finally, for dominance, the changes are represented by the size of the SAM, whereby the larger the manikin, the greater is the respondent’s control of the situation. The participants score each dimension on a scale between 1 and 9, with higher values indicating higher levels of pleasure, arousal and dominance. In our study, the participants received the following instructions for the SAM: *for each of the images, you will be asked to make an assessment and indicate how you feel based on the following criteria*. The SAM images and the detailed instructions we used can be consulted in the Supplementary material.

The Spanish version of the TYM is a self-administered cognitive screening test designed to identify Alzheimer’s disease and mild cognitive impairment. Its sensitivity is 0.86 and its specificity is 0.88 at the cut-off point of ≤40/50 to identify individuals with dementia (Ferrero-Arias and Turrión-Rojo, 2016).

### 2.4 Procedure

The participants were recruited using non-probabilistic convenience sampling. The group of young adults was selected from among students of the Medical School in Albacete, who voluntarily accepted to participate in the study. The group of older adults were recruited from among the social contacts of other students at the same educational institution.

Before forming part of our sample, potential participants were informed of the aims of the study, what was involved in their participation, and the entirely voluntary nature of their taking part. Once they were selected for the study, data were collected using a self-administered paper and pencil booklet.

Participants were exposed to five photos from the IAPS containing the following scenes: a family dining in a restaurant (IAPS photo 2,299 - Family), an adult and child at the beach (IAPS photo 5,831 - Seagulls), a girl sitting on the floor, covering her face (IAPS photo 9,041 - ScaredChild), a boy with a black eye (IAPS photo 2,345 - BlackEye), and a woman with a child in her arms (IAPS photo 2,312 - Mother). The runs were counterbalanced so that the order of presentation changed between participants. The different combinations led us to establish 10 conditions. The SAM questionnaire was on the same page as the photo, with participants being asked to rate the level of pleasure, arousal and dominance generated by their viewing the photo. The IAPS normative scores for these photos are included in Table 1 (Lang et al., 2008). Taking into account that valence can range from 1 to 9, we considered the photos of the family and the adult and child at the beach as positive, those of the girl covering her face and the boy with the black eyes as negative, and the photo of the woman with a child in her arms as neutral.

**Table 1:** Descriptive statistics for the selected IAPS pictures based on normative ratings (Lang et al., 2008).

Description	Slide *n*° (picture set)	Valence	Arousal	Dominance
		*M* (SD)	*M* (SD)	*M* (SD)
Family	2,299 (13)	7.27 (1.53)	3.95 (2.22)	6.18 (1.93)
Seagulls	5,831 (9)	7.63 (1.15)	4.43 (2.49)	6.46 (2.12)
ScaredChild	9,041 (9)	2.98 (1.58)	4.64 (2.26)	4.38(2.34)
BlackEye	2,345 (17)	2.26 (1.46)	5.50 (2.34)	3.96 (2.02)
Mother	2,312 (12)	3.71 (1.64)	4.02 (1.66)	4.72 (1.86)

On the next page of the book, participants found the following question: when looking at the above picture, did you think of a specific memory (something that happened at a specific time and day) from your childhood (yes/no). If you answer yes, please write the memory down.

This study was carried out in accordance with the recommendations of Agreement 06/2016 of the Clinical Research Ethics Committee (CREC). The protocol was approved by the Clinical Research Ethics Committee of the Gerencia de Atención Integrada de Albacete. All participants gave their written informed consent in accordance with the Declaration of Helsinki.

### 2.5 Statistical analysis

To assess the between-group differences (young and older adults) in emotional reaction to the visual stimuli and the number of memories retrieved, we conducted comparisons of means using the t-student test and Cohen’s d. The same statistics were used to evaluate the differences in valence, arousal and dominance for the stimuli that elicited a memory and those that did not. The proportion of memories generated by each type of cue in each group was compared by means of a chi-squared test. Finally, the variables associated with memory retrieval were analyzed using binary logistic regression, with a memory having been generated as the dichotomous dependent variable. The independent variables were age, sex, years of education and type of photo (positive, negative or neutral). After assessing the results of the saturated model, the data were cleaned, implementing a mixed forward-backward system, using the ACI as the model section criterion.

## 3 Results

As regards the responses to the five affective pictures (IAPS) in valence, arousal and dominance, we only found statistically significant differences for picture 2,345 (BlackEye), with the valence (pleasure) being higher and the arousal lower in the older adult group, and for picture 2312 (Mother) the valence (pleasure) being higher in the older adult group (Table 2).

**Table 2:** Emotional valence, arousal and dominance by affective picture and age group.

IAPS	Young adults		Older adults		*t*	*p*	Cohen’s *d*
	*M*	SD	*M*	SD			
Family (2299)							
Valence	8.31	1.06	8.30	1.12	0.12	0.908	0.01
Arousal	3.62	2.41	3.68	2.66	−0.22	0.830	−0.02
Dominance	6.89	2.13	6.77	2.31	0.46	0.644	0.05
Seagulls (5831)							
Valence	8.44	1.11	8.36	0.10	0.65	0.517	0.08
Arousal	2.73	2.13	3.11	2.49	−1.51	0.133	−0.16
Dominance	7.04	2.03	7.02	2.18	0.10	0.922	0.01
ScaredChild (9041)							
Valence	1.97	1.15	2.30	1.83	−1.95	0.053	−0.22
Arousal	6.43	1.78	6.52	2.05	−0.44	0.660	−0.05
Dominance	4.50	2.17	4.65	2.50	−0.57	0.567	−0.06
BlackEye (2345)							
Valence	1.42	0.80	2.08	2.01	−3.82	<0.001	−0.43
Arousal	7.13	1.99	6.55	2.27	2.43	0.016	0.27
Dominance	4.41	2.60	4.79	2.55	−1.32	0.189	−0.15
Mother (2312)							
Valence	3.43	2.04	4.57	2.52	−4.46	<0.001	−0.50
Arousal	5.18	1.90	5.34	2.34	−0.68	0.496	−0.08
Dominance	5.27	2.17	5.33	2.34	−0.24	0.813	−0.01

Regarding the number of memories elicited by the photos, we divided the results into 4 variables: total number of memories with all five stimuli (two positive pictures, two negative pictures and one neutral picture), number of memories on the two positive stimuli, number of memories on the two negative stimuli, and number of memories on the neutral stimulus. The results revealed statistically significant differences in all cases, with a larger number of memories in the group of young adults, with the exception of the neutral photo of the mother (Table 3).

**Table 3:** Comparison (young adults vs older adults) of the number of memories elicited by the affective pictures (IAPS).

IAPS	Young adults		Older adults		*t*	*p*	Cohen’s *d*
	*M*	SD	*M*	SD			
Total stimuli	2.00	1.08	1.44	1.24	4.35	<0.001	0.48
Positive stimuli	1.39	0.71	0.91	0.83	5.61	<0.001	0.62
Negative stimuli	0.44	0.62	0.28	0.54	2.56	0.011	0.28
Neutral stimulus	0.17	0.380	0.26	0.44	−1.88	0.062	−0.22

Table 4 shows the differences in proportions between the age groups as regards the memories generated for each of the stimuli.

**Table 4:** Frequencies and comparisons of proportions between the two age groups according to memories generated for each photo.

IAPS	Young adults		Older adults			
	*n*	%	*n*	%	*χ^2^*	*p*
Family (2299)	114	67.9	81	50.9	9.71	0.002
Seagulls (5831)	119	70.8	63	39.6	32.24	<0.001
ScaredChild (9041)	44	26.2	20	12.6	9.62	0.002
BlackEye (2345)	30	17.9	24	15.1	0.45	0.501
Mother (2312)	29	17.3	41	25.8	3.53	0.060

As for the relationship between the levels of valence, arousal and dominance, and the generation (or not) of a memory, we found significant differences between the stimuli that elicited a memory and those that did not (Table 5). Specifically, the images that elicited a memory were rated with higher valence (pleasure) and dominance, and with lower arousal.

**Table 5:** Differences in valence, arousal and dominance between the stimuli that elicited a memory and those that did not.

Stimulus	Memory		No memory		*t*	*p*	Cohen’s *d*
	*M*	SD	*M*	SD			
Valence	6.61	3.09	4.14	3.12	−14.95	<0.001	0.80
Arousal	4.16	2.75	5.43	2.58	8.82	<0.001	−0.48
Dominance	6.24	2.41	5.40	2.55	−6.30	<0.001	0.34

We estimated a binary logistic regression model to assess the variables associated with memory generation. Table 6 shows the results of this model. The variables associated with generating a memory were age (OR: 0.98), years of education (OR: 0.97) and type of photo (positive versus neutral) (OR: 5.47).

**Table 6:** Logistic regression model for the variables associated with memory generation.

Variable	OR	SD	Wald	*p*	95% CI
Constant	0.64	0.28	−1.57	0.117	[0.37, 1.12]
Age	0.98	0.003	−6.79	<0.001	[0.98, 0.99]
Education (years)	0.97	0.02	−2.13	0.033	[0.94, 1.00]
Type of photo					
Positive-neutral	5.47	0.17	10.03	<0.001	[3.95, 7.68]
Negative-neutral	0.97	0.18	−0.18	0.855	[0.68, 1.39]

## 4 Discussion

The main aim of this study was to analyze the age differences in emotional reactivity and autobiographical memory induction using childhood-related photos from the IAPS. The results revealed differences between young and older adults in both emotional reactivity and autobiographical memory retrieval.

Concerning emotional reactivity, the results for valence and arousal after exposure to the pictures revealed differences on two of the five photos presented: on the image “(BlackEye),” which presented a more positive valence and lower arousal in the older adult group, and on the image “Mother,” which also showed a more positive valence in the group of older adults. These findings can be considered consistent with the SST, under which a tendency toward positivity would be expected as age increases (Mather and Carstensen, 2005). These authors suggest that strategic processes in attention and memory related to emotions in older people may play an important role in the age-related differences in this positivity bias. In our study, both in the case of both a negative photo (BlackEye) and a neutral photo (Mother), the rating was more positive in the group of older adults. In this sense, some studies have reported contrasting conclusions to ours, finding that valence and arousal in response to IAPS stimuli show a tendency to more extreme ratings with age, such that older adults see the positive stimuli as more positive than young adults and the negative ones as more negative (Grühn and Scheibe, 2008), which contrasts with our findings. This difference could be explained by the procedure. While in our study we asked the participants to think about how the images made them feel, in Grühn and Scheibe’s work, the question was focused on classifying the valence and arousal of the image. In any event, the differences in ratings between the young and older adults may be related to the meaning each group attached to the stimulus, which may have been perceived as more, or less, pleasant by the different age groups (Backs et al., 2005). It could be the case that the difference in the response to the BlackEye image is related to the difference in attitudes to child abuse in the two generations. Similarly, the Mother image might have been assigned a more positive valence by the older generation because the mother depicted in the image is wearing clothes that may have resonated more with them.

We found no age-related differences for the other photos. Previous studies have reported a similar tendency, with no statistically significant differences found between ages on valence and arousal generated by IAPS stimuli (Ueno et al., 2019).

The number of autobiographical memories the participants retrieved in response to exposure to the photos was, as expected, higher among the young adults for both the positive and negative stimuli. As reported in the literature, when groups of young and older adults are asked to recall specific autobiographical memories, the number retrieved is higher in participants of a lower age (Ros et al., 2018b; Mair et al., 2021). Similarly, the number of spontaneous memories is typically larger in young individuals. In a study that assessed both types of memories (voluntary and involuntary), the number of memories was lower in older participants in both cases. In addition, the involuntary memories were found to be more positive in the group of older persons (Schlagman et al., 2009). Generally speaking, older adults participate less actively in the direct retrieval of autobiographical memories (Wank et al., 2021).

The photos that more directly generated autobiographical memories were those with a more positive valence, lower levels of arousal (greater relaxation) and greater emotional control. Such findings are in line with those of Clark et al., whose study used a film as a stimulus, after the viewing of which the participants’ involuntary autobiographical memories were assessed. Although this study was different from ours in the sense that the memories of the film were recorded in a diary over the week following the viewing, the authors found that a positive emotional reaction to the stimulus predicted the frequency of the subsequent positive involuntary autobiographical memories (Clark et al., 2013). In this sense, we can refer to the functional avoidance hypothesis, which postulates that the reduction in the specificity of autobiographical memory could be the result of an affect regulation strategy, such that it would minimize the psychological consequences following an unpleasant experience (Hermans et al., 2005; Williams, 2006; Williams et al., 2007). Reducing the specificity of negative memories may prevent individuals from re-experiencing the associated painful emotions (Debeer et al., 2012). This may explain why negative stimuli might fail to elicit specific autobiographical memories.

It is worth noting here that the percentage of memories elicited by the stimuli in our study cannot be considered high, especially for the negative photos and the neutral image, ranging, in these cases, between around 12 and 25%. This is related to the study procedure, in which the participants were not instructed to retrieve an autobiographical memory. The elicitation method may also play a role in these results, given that pictorial cues have been experimentally shown to be less effective in eliciting involuntary autobiographical memories than other methods, such as verbal cues. They do, however, generate more vivid memories (Mazzoni et al., 2014). Other methods have also been used to elicit autobiographical memories, such as odors, which evoke less specific memories than visual conditions (Goddard et al., 2005; Miles and Berntsen, 2011), but more located in the first decade of life (<10 years), whereas memories associated with visual cues peak in early adulthood (11–20 years) (Willander and Larsson, 2006). Finally, this result could also be related to the theme of the negative images, which depicted potential child abuse. This may not have been the participants’ experience, and could be an explanation of the low number of memories both young and older participants reported.

In our logistical regression analysis, the most important variables associated with memory induction –as regards statistical significance- were age and the stimulus presented being positive (compared to neutral). As discussed, the older the individual, the more likely is the difficulty in retrieving a memory (Mair et al., 2021), and the induction of positive mood favors the retrieval of positive memories (Clark et al., 2013). In fact, having viewed a positively themed photo compared to a neutral one considerably increased the likelihood of eliciting an autobiographical memory in our study, with this variable presenting the highest OR in the regression analysis. This association between emotional reactivity and autobiographical memory has been corroborated in studies such as that by Kensinger et al., which concludes that memory recall for emotionally active stimuli is better than for neutral stimuli in older adults (Kensinger et al., 2002). In the context of aging, a positivity effect associated with episodic autobiographical memories has also been reported, given that older persons tend to rate this type of memory more positively than young adults (Rathbone et al., 2015). A more robust association has also been demonstrated between psychological well-being and memory sensitivity, defined as “the tendency of individuals to pay particular attention, and accord particular value to their autobiographical memory” (Toffalini et al., 2016).

## 5 Conclusion

In the present study, we found that the group of older adults presented higher ratings on pleasure or positive valence and lower ratings on arousal after viewing negative or neutral stimuli, although no significant differences were revealed in the case of positive stimuli. The number of memories was higher in the younger adults, in the case of both positive and negative images. The number of memories was also higher for younger adults in the photos that generated higher levels of pleasure, greater relaxation and greater emotional control. Of the variables that might be associated with the induction of involuntary autobiographical memories, the most noteworthy were age and the stimulus being positive. The study of the association between AM recovery and emotional reactivity is a field which calls for further research. The findings of the present study show that positive emotion induction facilitates the retrieval of specific positive autobiographical memories in both age groups Therefore, this technique could be incorporated as a therapeutic strategy in the field of clinical psychology.

## 6 Limitations and future research

As a limitation of the study, we could consider that for the evaluation of involuntary autobiographical memories, we asked the participants, after each photo, whether any memory came to mind. Despite not having received explicit instructions to think about personal memories, after having seen the first few photos, we could consider that the participants already expected they would be asked about this topic, and this could have affected the memories, in the sense of not being completely involuntary. Another limitation of the study is related to the place and time of the data collection. The participants received instructions on how to complete the questionnaires and were able to do so at a place and time of their choice. These instructions included the need to find a quiet, distraction-free place and time.

In future research, a direct comparison of age-related involuntary and voluntary retrieval of autobiographical memories is necessary to determine how age-related involuntary retrieval differs from voluntary retrieval of autobiographical memories associated with IAPS pictures. It would also be interesting to assess the induction of involuntary autobiographical memories using other types of stimuli, such as verbal cues of film clips, in order to analyze differences according to the method implemented. Finally, it might be fruitful to delve deeper not only into whether a memory is retrieved, but also into the characteristics of the memories recalled, such as valence, specificity, the importance participants attach to such memories, or the time elapsed since the experience recalled.

## Data availability statement

The raw data supporting the conclusions of this article will be made available by the authors, without undue reservation.

## Ethics statement

The studies involving humans were approved by Clinical Research Ethics Committee of the Gerencia de Atención Integrada de Albacete. The studies were conducted in accordance with the local legislation and institutional requirements. The participants provided their written informed consent to participate in this study.

## Author contributions

BN: Conceptualization, Formal analysis, Investigation, Supervision, Writing – original draft, Writing – review & editing. MJ: Data curation, Investigation, Methodology, Supervision, Writing – review & editing, Writing – original draft. LF-A: Formal analysis, Investigation, Methodology, Writing – review & editing, Writing – original draft. MN: Data curation, Formal analysis, Writing – review & editing. AT-G: Data curation, Methodology, Writing – review & editing. MC: Conceptualization, Methodology, Writing – review & editing. LR: Conceptualization, Funding acquisition, Project administration, Writing – review & editing. JL: Conceptualization, Funding acquisition, Project administration, Supervision, Writing – review & editing.

## References

[ref1] Backs R. W. Da Silva S. P. Han K. (2005). A comparison of younger and older adults’ self-assessment manikin ratings of affective pictures. Exp. Aging Res. 31, 421–440. doi: 10.1080/03610730500206808 16147461

[ref2] Barry T. J. Gregory J. D. Latorre J. M. Ros L. Nieto M. Ricarte J. J. (2021). A multi-method comparison of autobiographical memory impairments amongst younger and older adults. Aging Ment. Health 25, 856–863. doi: 10.1080/13607863.2020.1729338, PMID: 32162531

[ref3] Berntsen D. Rubin D. C. (2002). Emotionally charged autobiographical memories across the life span: the recall of happy, sad, traumatic, and involuntary memories. Psychol. Aging 17, 636–652. doi: 10.1037//0882-7974.17.4.636, PMID: 12507360

[ref4] Bradley M. M. Lang P. J. (1994). Measuring emotion: the self-assessment manikin and the semantic differential. J. Behav. Ther. Exp. Psychiatry 25, 49–59. doi: 10.1016/0005-7916(94)90063-9, PMID: 7962581

[ref5] Britton J. C. Taylor S. F. Sudheimer K. D. Liberzon I. (2006). Facial expressions and complex IAPS pictures: common and differential networks. NeuroImage 31, 906–919. doi: 10.1016/j.neuroimage.2005.12.050, PMID: 16488159

[ref6] Carretero L. M. Latorre J. M. Fernández D. Barry T. J. Ricarte J. J. (2020). Effects of positive personal and non-personal autobiographical stimuli on emotional regulation in older adults. Aging Clin. Exp. Res. 32, 157–164. doi: 10.1007/s40520-019-01147-0, PMID: 30805866 PMC6974493

[ref7] Carstensen L. L. (2006). The influence of a sense of time on human development. Science 312, 1913–1915. doi: 10.1126/science.1127488, PMID: 16809530 PMC2790864

[ref8] Charles S. T. Mather M. Carstensen L. L. (2003). Aging and emotional memory: the forgettable nature of negative images for older adults. J. Exp. Psychol. Gen. 132, 310–324. doi: 10.1037/0096-3445.132.2.310, PMID: 12825643

[ref9] Clark I. A. Mackay C. E. Holmes E. A. (2013). Positive involuntary autobiographical memories: you first have to live them. Conscious. Cogn. 22, 402–406. doi: 10.1016/j.concog.2013.01.008, PMID: 23416539 PMC3675682

[ref10] Conway M. A. (2005). Memory and the self. J. Mem. Lang. 53, 594–628. doi: 10.1016/j.jml.2005.08.005

[ref11] Conway M. A. Pleydell-Pearce C. W. (2000). The construction of autobiographical memories in the self-memory system. Psychol. Rev. 107, 261–288. doi: 10.1037/0033-295x.107.2.261, PMID: 10789197

[ref12] Debeer E. Raes F. Claes S. Vrieze E. Williams J. M. G. Hermans D. (2012). Relationship between cognitive avoidant coping and changes in overgeneral autobiographical memory retrieval following an acute stressor. J. Behav. Ther. Exp. Psychiatry 43, S37–S42. doi: 10.1016/j.jbtep.2011.04.002, PMID: 23200429

[ref13] El Haj M. Kapogiannis D. Antoine P. (2020). The picture of the past: pictures to cue autobiographical memory in Alzheimer’s disease. J. Clin. Exp. Neuropsychol. 42, 914–923. doi: 10.1080/13803395.2020.1825636, PMID: 33003989 PMC9988368

[ref14] Fernández-Aguilar L. Latorre J. M. Martínez-Rodrigo A. Moncho-Bogani J. V. Ros L. Latorre P.. (2020). Differences between young and older adults in physiological and subjective responses to emotion induction using films. Sci. Rep. 10:14548. doi: 10.1038/s41598-020-71430-y, PMID: 32883988 PMC7471684

[ref15] Fernández-Aguilar L. Navarro-Bravo B. Ricarte J. Ros L. Latorre J. M. (2019). How effective are films in inducing positive and negative emotional states? A meta-analysis. PLoS One 14:e0225040. doi: 10.1371/journal.pone.0225040, PMID: 31751361 PMC6872151

[ref16] Fernández-Pérez D. Ros L. Latorre J. M. (2023). The role of the personal relevance of images in retrieving autobiographical memories for emotion regulation: a randomized controlled trial study. Curr. Psychol. doi: 10.1007/s12144-023-04582-5

[ref17] Ferrero-Arias J. Turrión-Rojo M. Á. (2016). Validation of a Spanish version of the test your memory. Neurologia (Barcelona, Spain) 31, 33–42. doi: 10.1016/j.nrl.2013.12.009, PMID: 24480594

[ref18] Fivush R. (2011). The development of autobiographical memory. Annu. Rev. Psychol. 62, 559–582. doi: 10.1146/annurev.psych.121208.131702 20636128

[ref19] Goddard L. Pring L. Felmingham N. (2005). The effects of cue modality on the quality of personal memories retrieved. Memory (Hove, England) 13, 79–86. doi: 10.1080/09658210344000594, PMID: 15724909

[ref20] Gould O. N. Dixon R. A. (1993). How we spent our vacation: collaborative storytelling by young and old adults. Psychol. Aging 8, 10–17. doi: 10.1037//0882-7974.8.1.10, PMID: 8461106

[ref21] Gruber J. Harvey A. G. Purcell A. (2011). What goes up can come down? A preliminary investigation of emotion reactivity and emotion recovery in bipolar disorder. J. Affect. Disord. 133, 457–466. doi: 10.1016/j.jad.2011.05.009 21665287 PMC3293100

[ref22] Grühn D. Scheibe S. (2008). Age-related differences in valence and arousal ratings of pictures from the international affective picture system (IAPS): do ratings become more extreme with age? Behav. Res. Methods 40, 512–521. doi: 10.3758/brm.40.2.512 18522062

[ref23] Hackländer R. P. M. Janssen S. M. J. Bermeitinger C. (2019). An in-depth review of the methods, findings, and theories associated with odor-evoked autobiographical memory. Psychon. Bull. Rev. 26, 401–429. doi: 10.3758/s13423-018-1545-3, PMID: 30406397

[ref24] Hermans D. Defranc A. Raes F. Williams J. M. G. Eelen P. (2005). Reduced autobiographical memory specificity as an avoidant coping style. Br. J. Clin. Psychol. 44, 583–589. doi: 10.1348/014466505X53461, PMID: 16368035

[ref25] Jakubowski K. Belfi A. M. Kvavilashvili L. Ely A. Gill M. Herbert G. (2023a). Comparing music- and food-evoked autobiographical memories in young and older adults: a diary study. Br. J. Psychol. 114, 580–604. doi: 10.1111/bjop.12639, PMID: 36779290 PMC10363233

[ref26] Jakubowski K. Walker D. Wang H. (2023b). Music cues impact the emotionality but not richness of episodic memory retrieval. Memory (Hove, England) 31, 1259–1268. doi: 10.1080/09658211.2023.2256055 37679863

[ref27] Jayaro C. De la Vega I. Díaz-Marsá M. Montes A. Carrasco J. L. (2008). The use of the international affective picture system for the study of affective dysregulation in mental disorders. Actas Esp. Psiquiatr. 36, 177–182. PMID: 18478458

[ref28] Kensinger E. A. Brierley B. Medford N. Growdon J. H. Corkin S. (2002). Effects of normal aging and Alzheimer’s disease on emotional memory. Emotion 2, 118–134. doi: 10.1037/1528-3542.2.2.118 12899186

[ref29] Kim E. S. Kim H. E. Kim J.-J. (2020). The neural influence of autobiographical memory related to the parent-child relationship on psychological health in adulthood. PLoS One 15:e0231592. doi: 10.1371/journal.pone.0231592, PMID: 32282812 PMC7153857

[ref30] Lang P. J. Bradley M. M. Cuthbert B. N. (1998). International affective pictures system (IAPS): Digitized photographs, instruction manual and affective ratings (IAPS). Gainesville, FL: University of Florida.

[ref31] Lang P. J. Bradley M. M. Cuthbert B. N. (2008). International affective picture system (IAPS): Affective ratings of pictures and instruction manual. Technical Report A-8. Gainesville, FL: University of Florida.

[ref32] Laureanti R. Bilucaglia M. Zito M. Circi R. Fici A. Rivetti F.. (2020). *Emotion assessment using machine learning and low-cost wearable devices*. Annual International Conference of the IEEE Engineering in Medicine and Biology Society. IEEE Engineering in Medicine and Biology Society. Annual International Conference, 2020, pp. 576–579. 10.1109/EMBC44109.2020.9175221 33018054

[ref33] Löckenhoff C. E. Carstensen L. L. (2004). Socioemotional selectivity theory, aging, and health: the increasingly delicate balance between regulating emotions and making tough choices. J. Pers. 72, 1395–1424. doi: 10.1111/j.1467-6494.2004.00301.x, PMID: 15509287

[ref34] Mair A. Poirier M. Conway M. A. (2021). Age effects in autobiographical memory depend on the measure. PLoS One 16:e0259279. doi: 10.1371/journal.pone.0259279, PMID: 34714869 PMC8555790

[ref35] Mather M. Carstensen L. L. (2005). Aging and motivated cognition: the positivity effect in attention and memory. Trends Cogn. Sci. 9, 496–502. doi: 10.1016/j.tics.2005.08.005, PMID: 16154382

[ref36] Mazzoni G. Vannucci M. Batool I. (2014). Manipulating cues in involuntary autobiographical memory: verbal cues are more effective than pictorial cues. Mem. Cogn. 42, 1076–1085. doi: 10.3758/s13421-014-0420-3, PMID: 24871426

[ref37] Miles A. N. Berntsen D. (2011). Odour-induced mental time travel into the past and future: do odour cues retain a unique link to our distant past? Memory (Hove, England) 19, 930–940. doi: 10.1080/09658211.2011.613847, PMID: 22032615

[ref38] Moltó J. Montañés S. Poy R. Segarra P. Pastor M. C. Tormo M. P.. (1999). Un nuevo método para el estudio experimental de las emociones: El International Affective Picture System (IAPS). Adaptación española. Rev. Psicol. Gen. Apl. 52, 55–87.

[ref39] Moltó J. Segarra P. López R. Esteller A. Fonfría A. Pastor M. C.. (2013). Adaptación española del “International Affective Picture System” (IAPS). Tercera parte. Anal. Psicol. 29, 965–984. doi: 10.6018/analesps.29.3.153591

[ref40] Munawar K. Kuhn S. K. Haque S. (2018). Understanding the reminiscence bump: a systematic review. PLoS One 13:e0208595. doi: 10.1371/journal.pone.0208595, PMID: 30533033 PMC6289446

[ref41] Rathbone C. J. Holmes E. A. Murphy S. E. Ellis J. A. (2015). Autobiographical memory and well-being in aging: the central role of semantic self-images. Conscious. Cogn. 33, 422–431. doi: 10.1016/j.concog.2015.02.017, PMID: 25781408

[ref42] Reed A. E. Chan L. Mikels J. A. (2014). Meta-analysis of the age-related positivity effect: age differences in preferences for positive over negative information. Psychol. Aging 29, 1–15. doi: 10.1037/a0035194, PMID: 24660792

[ref43] Ricarte J. Ros L. Serrano J. P. Martínez-Lorca M. Latorre J. M. (2016). Age differences in rumination and autobiographical retrieval. Aging Ment. Health 20, 1063–1069. doi: 10.1080/13607863.2015.1060944, PMID: 26134094

[ref44] Ros L. Latorre J. M. Serrano J. P. Ricarte J. J. (2017). Overgeneral autobiographical memory in healthy young and older adults: differential age effects on components of the capture and rumination, functional avoidance, and impaired executive control (CaRFAX) model. Psychol. Aging 32, 447–459. doi: 10.1037/pag0000175, PMID: 28594191

[ref45] Ros L. Romero D. Ricarte J. J. Serrano J. P. Nieto M. Latorre J. M. (2018). Measurement of overgeneral autobiographical memory: psychometric properties of the autobiographical memory test in young and older populations. PLoS One 13:e0196073. doi: 10.1371/journal.pone.0196073 29672583 PMC5908191

[ref46] Rubin D. C. Berntsen D. (2009). The frequency of voluntary and involuntary autobiographical memories across the life span. Mem. Cogn. 37, 679–688. doi: 10.3758/37.5.679, PMID: 19487759 PMC3044938

[ref47] Schlagman S. Kliegel M. Schulz J. Kvavilashvili L. (2009). Differential effects of age on involuntary and voluntary autobiographical memory. Psychol. Aging 24, 397–411. doi: 10.1037/a0015785 19485657

[ref48] St-Laurent M. Abdi H. Burianová H. Grady C. L. (2011). Influence of aging on the neural correlates of autobiographical, episodic, and semantic memory retrieval. J. Cogn. Neurosci. 23, 4150–4163. doi: 10.1162/jocn_a_00079, PMID: 21671743 PMC3262842

[ref49] Toffalini E. Borella E. Cornoldi C. De Beni R. (2016). The relevance of memory sensitivity for psychological well-being in aging. Qual. Life Res. Int. J. Qual. Life Asp. Treat. Care Rehab. 25, 1943–1948. doi: 10.1007/s11136-016-1231-8, PMID: 26810180

[ref50] Toledano-González A. Romero-Ayuso D. Fernández-Pérez D. Nieto M. Ricarte J. J. Navarro-Bravo B.. (2023). Effects of the use of autobiographical photographs on emotional induction in older adults: a systematic review. Psychol. Res. 87, 988–1011. doi: 10.1007/s00426-022-01712-9, PMID: 35859072

[ref51] Ueno D. Masumoto K. Sato S. Gondo Y. (2019). Age-related differences in the international affective picture system (IAPS) valence and arousal ratings among Japanese individuals. Exp. Aging Res. 45, 331–345. doi: 10.1080/0361073X.2019.1627493, PMID: 31216947

[ref52] Van den Hoven E. Orth D. Zijlema A. (2021). Possessions and memories. Curr. Opin. Psychol. 39, 94–99. doi: 10.1016/j.copsyc.2020.08.014 32932200

[ref53] Wank A. A. Andrews-Hanna J. R. Grilli M. D. (2021). Searching for the past: exploring the dynamics of direct and generative autobiographical memory reconstruction among young and cognitively normal older adults. Mem. Cogn. 49, 422–437. doi: 10.3758/s13421-020-01098-2, PMID: 32965620 PMC8609962

[ref54] Wei M. Roodenrys S. Miller L. Barkus E. (2020). Complex scenes from the international affective picture system (IAPS). Exp. Psychol. 67, 194–201. doi: 10.1027/1618-3169/a000488, PMID: 32900297 PMC8210657

[ref55] Willander J. Larsson M. (2006). Smell your way back to childhood: autobiographical odor memory. Psychon. Bull. Rev. 13, 240–244. doi: 10.3758/bf03193837, PMID: 16892988

[ref56] Williams J. M. G. (2006). Capture and rumination, functional avoidance, and executive control (CaRFAX): three processes that underlie overgeneral memory. Cognit. Emot. 20, 548–568. doi: 10.1080/02699930500450465, PMID: 26529222

[ref57] Williams J. M. G. Barnhofer T. Crane C. Herman D. Raes F. Watkins E.. (2007). Autobiographical memory specificity and emotional disorder. Psychol. Bull. 133, 122–148. doi: 10.1037/0033-2909.133.1.122, PMID: 17201573 PMC2834574

[ref58] Williams J. M. Healy H. G. Ellis N. C. (1999). The effect of imageability and predicability of cues in autobiographical memory. Q. J. Exp. Psychol. A 52, 555–579. doi: 10.1080/713755828, PMID: 10504900

