# Supplementary material for: Effects of affectively-loaded childhood-related photos from the IAPS on the induction of involuntary autobiographical memories in young and older adults
Source: Front Psychol. 2024 Jan 11;14:1266758. doi: 10.3389/fpsyg.2023.1266758 (PMC10811954; doi:10.3389/fpsyg.2023.1266758)
Supplement: Supplementary file 1 [file Image_1.pdf]

SUPPLEMENTARY INFORMATION

INSTRUCTIONS FOR ADMINISTRATION OF SAM QUESTIONNAIRE IN OUR STUDY

“On the following pages, a series of images are presented. For each of the images, you will be asked to make an assessment and indicate how you feel based on the following criteria:
Level of unpleasantness or pleasantness of the picture you have seen. You can score from 1, "very unpleasant", to 9, "very pleasant".

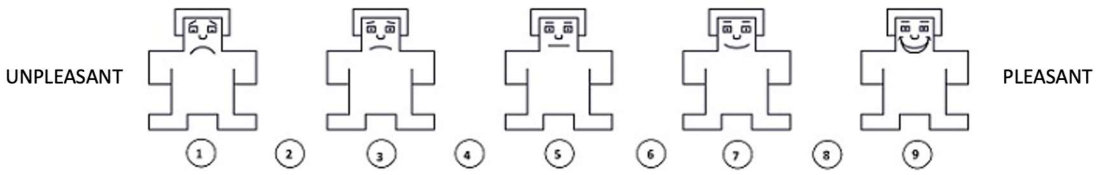

Level of activation produced by the picture you have seen. You can score from 1, "totally relaxed", to 9, "totally excited".

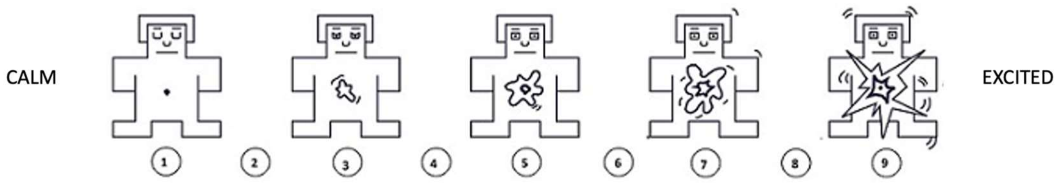

Emotional control you experienced when viewing the image. You can score from 1, "no control over my emotions", to 9, "total control over my emotions".

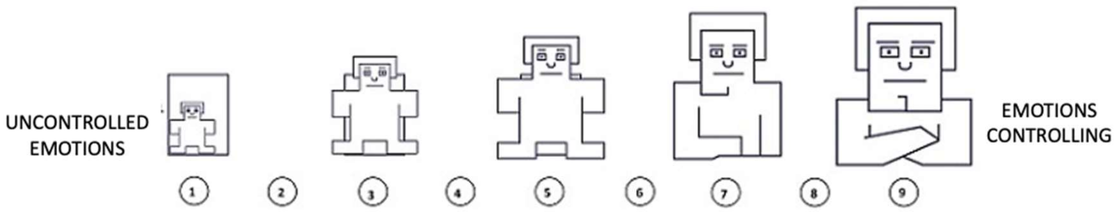

For example, if you found any of the images very pleasant, then you would mark number 9.

Each picture was presented on a separate page along with the measure of the three affective dimensions of the SAM questionnaire. Once this part had been answered, the questions about the memory were presented: "When looking at the above picture, did you think of a specific memory (something that happened at a specific time and day) from your childhood (yes/no). If you answer yes, please write the memory down:"

SELF ASSESSMENT MANIKIN © Peter J. Lang 1994
